# Epidemiology and distribution of gastrointestinal parasites in fattening pig farms in northern Italy

**DOI:** 10.1007/s00436-024-08320-z

**Published:** 2024-08-22

**Authors:** Carolina Allievi, Marco Valleri, Sergio Aurelio Zanzani, Alessandro Zanon, Michele Mortarino, Maria Teresa Manfredi

**Affiliations:** Department of Veterinary Medicine and Animal Sciences, Università Degli Studi Di Milano, Via Dell’Università, 6, 26900 Lodi, Italy

**Keywords:** Domestic pigs, Fattening cycle, Gastrointestinal parasites, Zoonosis

## Abstract

In Italy, pig breeding is characterised by intensive farms in which parasitic diseases often present a subclinical pattern, while being responsible for decreased animal welfare and great economic losses. The present study aimed to assess the prevalence of major parasites in pigs, and, for this purpose, 880 faecal samples of fattening pigs raised in 22 intensive farms located in northern Italy were collected in two different sampling sessions, at the beginning and end of the fattening cycle. For the detection of helminth eggs and coccidian oocysts, a quantitative flotation technique was used, whereas a conventional PCR was performed to confirm the identification of cestode eggs found by copromicroscopic analysis. Moreover, data regarding herd management were collected to assess risk factors for parasite occurrence. A total of 95 samples were positive for at least one parasitic taxon (10.8%); the most detected parasite was *Ascaris suum* (7.6%), followed by *Trichuris suis* (1.7%) and *Cystoisospora suis* (0.9%). Further, eggs with morphometric features compatible with those of *Hymenolepis diminuta* were detected in 16 samples (1.8%), and the analysis of sequences confirmed the identification of cestode eggs. Statistical analysis showed that large farms and those applying the all-in/all-out system were associated with a lower risk of nematode infection. This study provided data on prevalence and burden of gastrointestinal parasites in two different times of the fattening cycle. It was evidenced that endoparasites are persistent, albeit with low prevalences, and would need specific measures to reduce their effects on both animal health and productivity.

**Supplementary Information:**

The online version contains supplementary material available at 10.1007/s00436-024-08320-z.

## Introduction

In Italy, pig farms are concentrated in northern regions, especially in Lombardy, where most of the total national pig herd is raised (Gazzonis et al. 2018). The increase of intensive farms, characterised by big facilities, has led to improved hygiene and biosecurity practices through their large-scale application. Moreover, following the re-emergence of African Swine Fever worldwide, mandatory measures have been updated to prevent and control infectious diseases that can both lead to economic losses and be transmitted to humans (Alarcón et al. 2021; Giarratana et al. 2021). Thus, it was demonstrated that controlling the entry of people and vehicles into the farm, limiting the access of wild animals and cleaning the pens, as well as applying vaccinations against specific diseases and performing routine faecal analysis can promote substantial economic benefits (Laanen et al. 2013; Stygar et al. 2020; Alarcón et al. 2021; Pettersson et al. 2021a).

Porcine parasites are common in all production systems and widespread throughout the world, particularly, those with a direct life cycle. In pigs, infections by gastrointestinal parasites often show a subclinical pattern, which results in less attention paid to them by both breeders and veterinarians and are rarely included as causative or contributing agents for the differential diagnoses of gastrointestinal disorders. However, they may be responsible for diarrhoea, enteritis, and vomiting, predisposing to other diseases and causing reduced growth rate and feed conversion, altered fat distribution and the discarding of parasitised organs at slaughter, with a strong impact on host productivity (Worliczek et al. 2007; Kipper et al. 2011; Roepstorff et al. 2011; Symeonidou et al. 2020).

Another point to highlight is that some porcine parasites may pose a risk of infection for professionals involved in the food chain (e.g., farmers, veterinarians, and slaughterhouse workers) for their zoonotic potential, either by direct contact or exposure to contaminated environments (Nejsum et al. 2012; Zhou et al. 2012; Giarratana et al. 2021).

The update of pig welfare legislation has led to more intervention by health authorities, who encourage farmers to improve facilities, by, for example, adding manipulable rooting material which promotes animal welfare (Council Directive EC No. 2008/120). Nevertheless, this could lead to an increased risk of maintaining parasite cycles in farms (Pettersson et al. 2021b). At the farm level, other factors can influence the presence of gastrointestinal parasites, as the floor type, the presence of outdoor access, the use of bedding, and the type of production cycle, i.e. all-in/all-out systems or not (Joachim et al. 2001; Kochanowski et al. 2017). Moreover, the misuse of antiparasitic drugs, which includes the routine application without knowing the actual status of the herd, the use of the same active ingredient, and the possible administration of sub-therapeutic dosages, could increase the selection on resistance alleles in the parasite population, allowing more worms to survive the treatment and reducing animal welfare (Macrelli et al. 2019; Pettersson et al. 2021c).

The review of scientific literature revealed several shortcomings regarding the epidemiology and distribution of the main gastrointestinal parasites and associated risk factors in fattening intensive pig farms; indeed, only one study on the main endoparasites found in pigs raised in intensive systems (Marchesi 2009), and few surveys, related only to *Ascaris suum* detection at slaughter and by serology, were recently carried out in northern Italy (Scollo et al. 2017; Vismarra et al. 2023). Therefore, considering the relevance of pig farming in northern Italian regions, the main purposes of this cross-sectional study were to update the prevalence of major endoparasites in intensive pig farms located in this area, including the possible circulation of zoonotic parasites, and to investigate farm-level risk factors predisposing to parasitic infections.

## Materials and methods

### Sampling and data collection

**Fig. 1:**
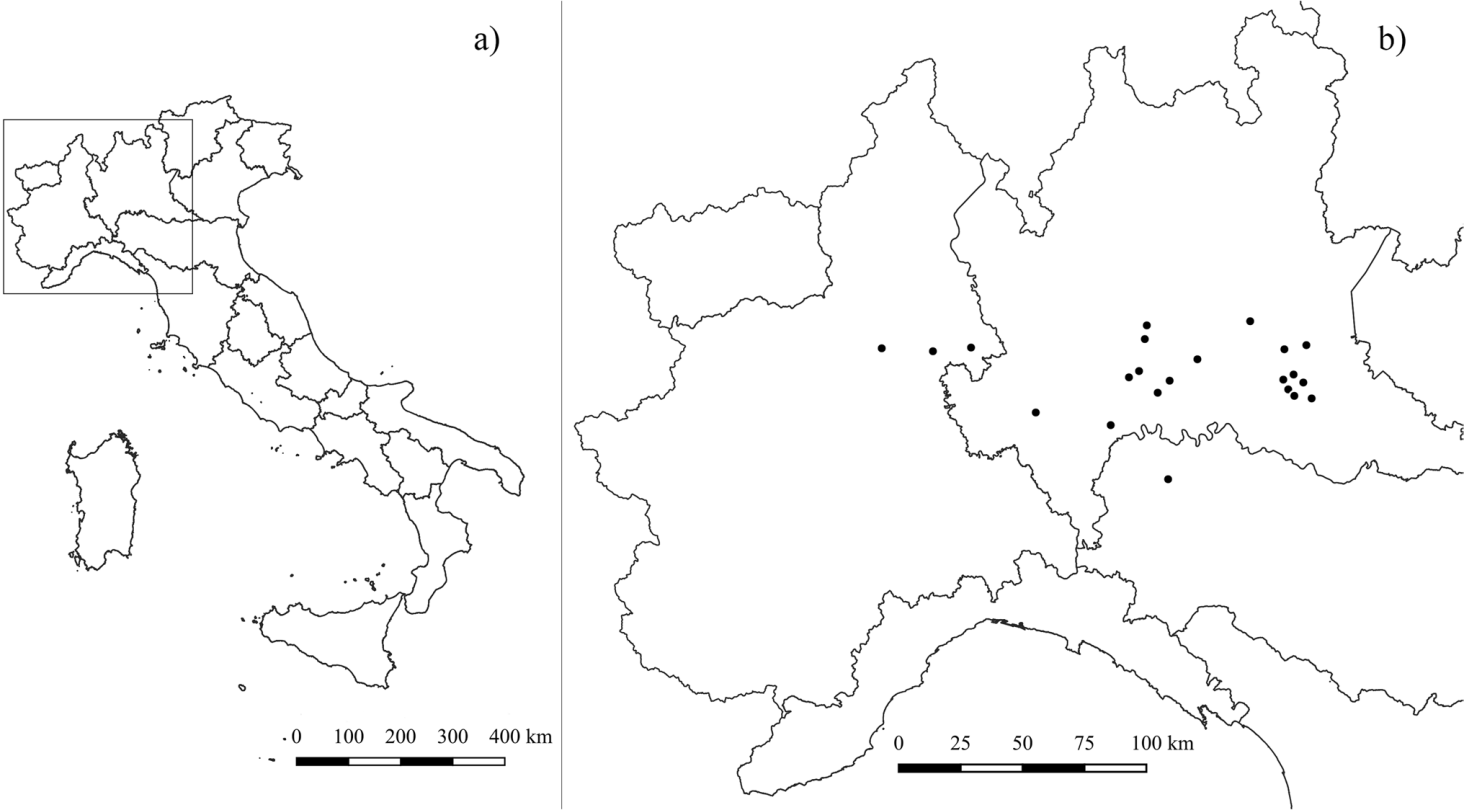
Spatial distribution of selected fattening pig farms in northern Italy using QGis (version 3.28.01 Firenze). (**a**) Black square = investigated area; (**b**) black lines = Italian regional boundaries, black dots = pig farms

The study was conducted in northern Italy: 22 fattening pig farms located in different regions, 18 in Lombardy, three in Piedmont and one in Emilia-Romagna, were included. Spatial distribution of sampled farms, according to different regions, is represented in Fig. 1. All farms were intensive fattening farms, where pigs were housed at 3 months of age weighing between 30 and 50 kg, and slaughtered at the age of nine months weighing between 160 and 180 kg.

The sampling was carried out in 2023, from April to October. A minimum sample size of 246 faecal samples was determined by Epitools Epidemiological Calculators (www.epitools.ausvet.com.au), considering a population of fattening pigs in northern Italy (including only fatteners present in the three selected regions: Lombardy = 1.191.288, Piedmont = 418.313, and Emilia-Romagna = 313.495) of about 2 million, a 20% expected prevalence, a 95% confidence level, and a 5% desired absolute precision (National Zootechnical Database, https://www.vetinfo.it/). Overall, 880 pigs, which were commercial hybrids of Landrace and Large White breeds, were sampled from 22 farms in two different sampling session: at time 1 (T1), i.e. at the beginning of the fattening cycle, and at time 2 (T2), just before slaughter. From each farm, 20 faecal samples were randomly selected from different pens (about four sampled animals for each pen) and collected in each sampling session (440 samples at T1 and 440 at T2) with a gloved hand from the rectal ampulla to avoid contamination. All sampled animals were raised in groups in different pens, consisting of 20 pigs, and the feed was dosed and administered twice a day, morning and evening respectively. Moreover, they were apparently healthy, with no clinical signs referable to the presence of gastrointestinal parasites. It is worth noting that in those farms where anthelmintic prophylaxis was regularly applied immediately after housing, all faecal samples collected at T1 were taken before the anthelmintic treatment. After collection, faecal samples were placed individually in plastic containers, labelled, and transferred to the laboratory, refrigerated at + 4 °C. Two aliquots from each sample were stored at − 20 °C for subsequent molecular analyses. Data on farm management, including farm size (< 1900 animals, ≥ 1900 animals), type of floor (full, slatted, mixed), outdoor access (yes/no), application of all-in/all-out system (yes/no), and application of antiparasitic treatment (yes/no) were collected by interviewing the farmer.

## Copromicroscopic and molecular analysis

Copromicroscopic analysis was carried out in the two days immediately following the collection by a quantitative flotation technique. For each sample, FLOTAC® dual technique, with an analytic sensitivity of two eggs/oocysts/larvae per gram (EPG/OPG/LPG) of faeces, was used (Cringoli et al. 2010). Two different flotation solutions, FS2 (sodium chloride, NaCl; s.g. = 1.200) and FS7 (zinc sulphate, ZnSO_4_; s.g. = 1.350), were separately employed to process all collected samples. The EPG/OPG were calculated for all nematodes and coccidia, while cestode infection was only evaluated by qualitative analysis. For samples positive for coccidian oocysts by copromicroscopic examination, the sporulation was induced on previously pelleted faecal material by placing it in a thermostat at 25 °C for at least 12 days, considering the different sporulation time of the genera *Cystoisospora* (1–2 days) and *Eimeria* (5–12 days). The identification of sporulated oocysts was performed using Sheather’s sugar solution (s.g. = 1.290) (Harleman and Meyer 1984; Joachim and Schwarz 2015; Joachim et al. 2018).

**Fig. 2:**
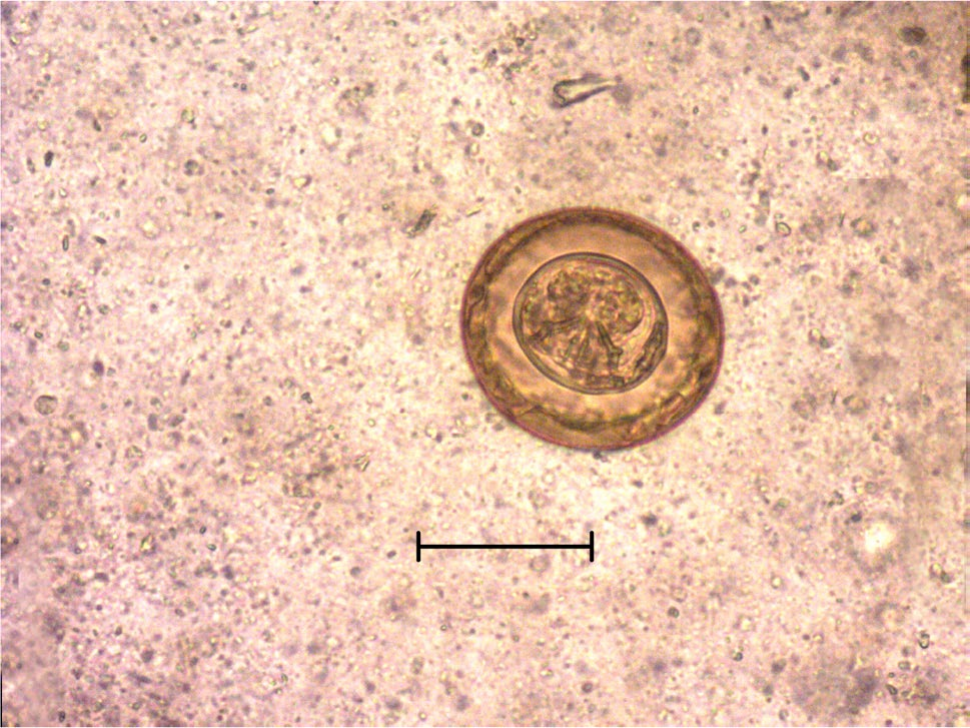
An egg of *Hymenolepis diminuta* found in pig faecal samples by light microscopy (400 × magnification) Scale bar: 50 µm

To identify cestode eggs (Fig. 2), genomic DNA was extracted from approximately 200 mg of faecal samples that tested positive for cestode eggs by FLOTAC® dual technique, using a commercial kit (QIAamp® Fast DNA Stool Mini Kit, QIAGEN, Hilden, Germany), following the manufacturer’s instructions. The extracted DNA concentration and purity were evaluated by the 260/280 nm ratio using the NanoDrop ND-1000 spectrophotometer (Nanodrop ND 1000, Thermo Scientific, Wilmington, DE, USA). Then, DNA samples were stored at − 20 °C until further processing. DNA samples were subjected to a conventional PCR amplifying nucleotide sequences of a 471 bp region of the mitochondrial NADH dehydrogenase 1 gene using universal primers for detection and identification of cestodes (Bowles and McManus 1993). The reactions were performed in a final volume of 50 μL, containing 5 μL of 10X DreamTaq Buffer including 20 mM of MgCl_2_ (Thermo Fisher Scientific, Life Technologies, Monza, MB, Italy), 5 μL of 2 mM dNTP Mix (Thermo Fisher Scientific, Life Technologies, Monza, MB, Italy), 1 μM of each primer (JB11, 5′-AGATTCGTAAGGGGCCTAATA-3′; and JB12, 5′-ACCACTAACTAATTCACTTTC-3′), 0.25 µl of DreamTaq DNA Polymerase 5U (Thermo Fisher Scientific, Life Technologies, Monza, MB, Italy), 32.75 μL of nuclease-free water (Sigma-Aldrich, Italy), and 5 μL of DNA samples (approximately 25–50 ng of genomic DNA). The PCR reactions were performed in a thermal cycler (Applied Biosystems SimpliAmp Thermal Cycler, Waltham, MA, USA). The reaction was performed with an initial denaturation step of 95 °C for 3 min, followed by 10 cycles of denaturation (30 s at 95 °C), annealing (35 s at 50 °C), and extension (30 s at 72 °C), followed by 30 cycles of denaturation (30 s at 95 °C), annealing (35 s at 48 °C), extension (30 s at 72 °C), and a final extension step (7 min at 72 °C). Positive and negative (no-template) controls were included in the run. PCR products were run on 1.5% agarose gel containing 0.05% ethidium bromide in TBE buffer electrophoresis and visualised under UV light on a transilluminator using a 500 bp DNA ladder (GeneRuler, Thermo Fisher Scientific, Life Technologies, Monza, MB, Italy) as a size standard. Bands of the expected size were excised from agarose gel and purified with a commercial kit (NucleoSpin® Gel and PCR Clean-up, Macherey–Nagel, Düren, Germany) following the manufacturer’s instructions. Then, purified PCR products were sent for bidirectional sequencing to a commercial service (Microsynth Seqlab, Göttingen, Germany). Electropherograms were checked, and consensus sequences were manually assembled. Sequences were compared to nucleotide sequences available in the GenBank database using the BLASTN program (https://blast.ncbi.nlm.nih.gov/, accessed on 3 September 2023) and then aligned each other using the Mega6 software (Tamura et al. 2013).

### Statistical analysis

**Fig. 3:**
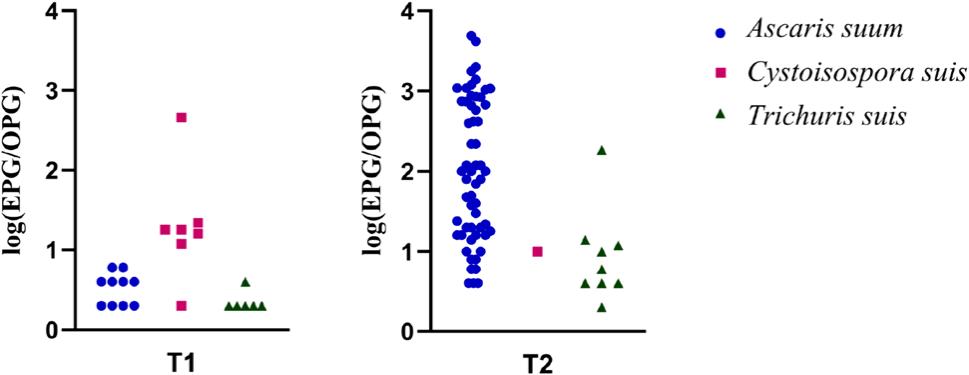
Logarithmic distribution of faecal EPG/OPG of detected nematodes and coccidia in positive samples according to each sampling session (T1 = beginning of fattening cycle and T2 = end of fattening cycle)

The status of each farm and animal (infected or non-infected) for helminths (*A. suum*, *Trichuris suis*, Hymenolepididae) and coccidia was determined by copromicroscopic analysis. A farm/animal was considered infected if at least one helminth egg or coccidian oocyst was observed. The rates of infected animals were calculated, and distributions of eggs or oocysts observed per gram of faeces analysed by considering the abundance and standard deviation with minimum and maximum excretion (Bush et al. 1997). The logarithmic distribution of faecal EPG/OPG of detected parasites in each sampling session was presented in Fig. 3 using Prisma GraphPad Version 10.1.0. (GraphPad Software, La Jolla California USA). Statistical analysis was carried out only on samples collected in the second sampling session (T2), and prevalence values of each parasite were associated with categorised management characteristics. Then, farm management data (farm size, type of floor, outdoor access, application of all-in/all-out system, application of antiparasitic treatment) were assessed as risk factors for parasite occurrence and introduced into generalised linear mixed models (GLMMs) as categorical independent variables, while parasite status (positive/negative) was introduced into the models as the dependent variable. In addition, positivity/negativity to the different parasites observed in the first sampling session (T1) in each farm was considered a variable influencing the positivity to parasites at T2. Each farm included in this study was considered a random intercept effect. The models that best explained parasite positivity were chosen by backward elimination and best Akaike’s information criterion (AIC). Variables showing a *p* value < 0.05 were considered statistically significant. Statistical analysis was performed using SPSS software (version 28.0.1.1, Chicago, IL, USA).

## Results

### Copromicroscopic and molecular analysis

Out of 880 individual faecal samples, 95 (10.8%, 95% CI: 8.8–13.0) were positive for at least one parasitic taxon; at the farm level, a total of 14 out of 22 farms were positive (63.6%, 95% CI: 40.7–82.8). Overall, *A. suum* was the most detected parasite, since it was found in 45.4% (95% CI: 24.4–67.8) of the farms and 7.6% (95% CI: 5.9–9.6) of the total number of individual samples (Table 1). *Trichuris suis* was found in six out of 22 farms (27.3%, 95% CI: 10.7–50.2), and 15 out of 880 fattening pigs (1.7%, 95% CI: 1.0–2.8) were positive. Unlike eggs/oocysts of the other parasites, detectable with both flotation solutions, eggs of *T. suis* were detected only when using zinc sulphate solution. Strongyle-type eggs and those of bronchopulmonary nematodes (*Metastrongylus* spp.) were not detected in any sample. All coccidian oocysts were classified, using the Sheather’s sugar solution, as belonging to *Cystoisospora suis*, while the genus *Eimeria* was not detected. *Cystoisospora suis* was sporadically found in both sampling sessions with a farm prevalence of 13.6% (95% CI: 3.1–35.1) and with 8 out of 880 positive samples (0.9%, 95% CI: 0.3–1.8). Finally, eggs of cestodes belonging to the family Hymenolepididae, with morphometric features compatible with those of *Hymenolepis diminuta*, were found in 16 out of 880 samples (1.8%, 95% CI: 1.0–2.9) (Fig. 2).

**Table 1:** Prevalence of gastrointestinal parasites in fattening pig farms in northern Italy according to the sampling session (T1 = beginning of fattening cycle and T2 = end of fattening cycle)

Detected parasites	Sampling session	Positive farms		Positive samples		EPG/OPG^a^	
		N°	Prevalence % (95% CI^b^)	N°	Prevalence % (95% CI)	Abundance (SD^c^)	Min–Max
*Ascaris suum*	T1	3/22	13.6 (3.1–35.1)	10/440	2.3 (1.1–4.1)	0.08 (0.6)	0–6
	T2	9/22	40.9 (20.7–63.6)	57/440	12.9 (10–16.5)	65.5 (370.3)	0–4900
	Total	10/22	45.4 (24.4–67.8)	67/880	7.6 (5.9–9.6)	32.8 (263.7)	0–4900
*Cystoisospora suis*	T1	3/22	13.6 (3.1–35.1)	7/440	1.6 (0.6–3.2)	1.2 (22)	0–460
	T2	1/22	4.5 (0.1–22.8)	1/440	0.2 (0.01–1.3)	0.02 (0.5)	0–10
	Total	3/22	13.6 (3.1–35.1)	8/880	0.9 (0.3–1.8)	0.6 (15.6)	0–460
*Trichuris suis*	T1	4/22	18.2 (5.2–40.3)	6/440	1.4 (0.5–2.9)	0.03 (0.3)	0–4
	T2	4/22	18.2 (5.2–40.3)	9/440	2 (0.9–3.8)	0.5 (8.9)	0–186
	Total	6/22	27.3 (10.7–50.2)	15/880	1.7 (1–2.8)	0.3 (6.3)	0–186
Hymenolepididae	T1	3/22	13.6 (3.1–35.1)	8/440	1.8 (0.8–3.5)	nd^d^	nd
	T2	5/22	22.7 (7.8–45.4)	8/440	1.8 (0.8–3.5)		
	Total	6/22	27.3 (10.7–50.2)	16/880	1.8 (1–2.9)		
**Total**	T1	10/22	45.4 (24.4–67.8)	26/440	5.9 (3.9–8.5)	nd	nd
	T2	12/22	54.5 (32.2–75.6)	69/440	15.7 (12.4–19.4)		
	Overall prevalence	14/22	63.6 (40.7–82.8)	95/880	10.8 (8.8–13)		

^a^Eggs per gram/oocysts per gram

^b^Confidence interval

^c^Standard deviation

^d^Not determined

Co-infections were observed in 11 out of 880 samples (1.2%, 95% CI: 0.6–2.2), and the most common associations were between *A. suum* and *T. suis* (5/880) and between *A. suum* and *H. diminuta* (5/880), while only one sample was positive for both *A. suum* and *C. suis*.

As for the faecal egg count of *A. suum*, it ranged from 4 EPG in the first sampling session (T1) to 4900 EPG at T2, with a mean of 32.8 EPG. As for *T. suis*, the mean of EPG was 0.3 with a maximum of 186 EPG in the second sampling session, while that of *C. suis* was 0.6 OPG, with a peak of oocyst excretion in the first sampling session, up to 460 OPG. The abundance and the minimum and maximum excretion of faecal EPG/OPG in each sampling session are shown in Table 1.

DNA extraction and conventional PCR were performed on 16 samples that tested positive for cestode eggs by copromicroscopic examination. All samples resulted positive for cestode DNA. Out of the 16 amplicons detected, all were sequenced and BLASTn analysis confirmed a 100% identity with *H. diminuta* (DNA reference sequences: LR536429, AP017664, HM149291, and NC_002767). Since no intraspecific nucleotide variations were observed between any of the obtained *H. diminuta* sequences, one representative partial sequence was submitted to GenBank under accession number PP982280.

### Comparison of farms at the beginning and end of fattening cycle

In eight out of 22 farms (36.3%, 95% CI: 17.2–59.3), no parasitic taxa were detected in either sampling session. Of the remaining 14 farms, 4 were negative at T1 and positive at T2, 2 were positive at T1 and negative at T2, and 8 were positive at both T1 and T2 (Suppl. Table S1). Particularly, T1-positive and T2-negative farms recorded only the presence of *C. suis*, while in T1-negative and T2-positive farms *A. suum*, *T. suis*, and *H. diminuta* were detected, but no *C. suis* oocyst were evidenced. The positive farms in both sampling sessions showed different parasitic taxa with a diverse distribution over time (Suppl. Table S1). Compared to the results of the first sampling session, higher intra-farm prevalences were recorded at T2, especially in farm 016, where 20 out of 20 samples were positive. An exception was farm 02, in which the prevalence was higher at T1, when both *A. suum* and *H. diminuta* were detected, while at T2 only one sample was positive, specifically to *H. diminuta* (Suppl. Table 1).

### Prevalence values, management practices, and risk factors

The descriptive results obtained at T2, associating the prevalence with categorised management characteristics, are highlighted in Suppl. Table S2. Farm positivity at T1 was also considered. Most of the recruited farms applied the all-in/all-out system (16/22) and treated pigs with the anthelmintic, after housing at the fattening site (18/22). In all farms that carried out the anthelmintic treatment, benzimidazoles were used. Outdoor access was evenly distributed in the two categories (yes/no); regarding the floor type, 12 farms had full floor, two mixed (full + slatted) and eight slatted. Overall, at T2, nine farms were positive for *A. suum*, five for *H. diminuta*, four for *T. suis*, and one for *C. suis*. For both *A. suum* and *T. suis*, a higher prevalence of infection was observed in animals raised in smaller farms, those where anthelmintic treatment was not performed and those without all-in/all-out system. At the farm level, 66.7% of herds positive for *A. suum* and *H. diminuta* and 50% of those positive for *T. suis* at T1 were also positive at T2; in contrast, *C. suis* oocysts were detected at T2 in only one sample from a single farm.

Data collected and categorised from each farm (Suppl. Table S2) were considered possible risk factors for parasite occurrence and introduced into GLMMs. The final GLMMs, by backward elimination and best AIC, are shown in Table 2: Large farms and those applying the all-in/all-out system were associated with a lower risk of *A. suum* and nematode (including both *A. suum* and *T. suis*) infections. As for *C. suis*, *H. diminuta*, and *T. suis* only, no significant predictors for infections were evidenced.

**Table 2:** Results of the final GLMMs of risk factors related to each detected parasite at T2 sampling session in fattening pig farms surveyed in northern Italy. Variables showing a *p* value < 0.05 were considered significant predictors of infection

Detected parasites	Variable	F^a^	Degrees of freedom	Category	Odds ratio (95% CI^b^)	*p* Value
*A. suum*	All-in/All-out	3.915	1	No	34 (1–1132)	0.048
				Yes (ref.)	1	
	Farm size	4.617	1	Large (≥ 1900 animals)	0.01 (0.03–0.2)	0.032
				Small (< 1900 animals) (ref.)	1	
Nematodes (*A. suum* + *T. suis*)	All-in/All-out	4.653	1	No	31.9 (1.4–748.3)	0.032
				Yes (ref.)	1	
	Farm size	5.931	1	Large (≥ 1900 animals)	0.01 (0–0.4)	0.015
				Small (< 1900 animals) (ref.)	1	

^a^Coefficient

^b^Confidence Interval

## Discussion

This study provided updated data on the circulation of gastrointestinal parasites and associated risk factors for infection in intensive pig farms in northern Italy. Overall, the prevalence values were low, although the use of a highly sensitive quantitative method allowed the detection of eggs/oocysts, even in case of low excretion (Cringoli et al. 2010).

*Ascaris suum* was the most detected parasite: 67 pigs out of 880 tested positive, with a prevalence of 7.6%, similar to that reported in other European countries, which ranged from 0.9% to 9%, depending on the diagnostic method used and the age of the animals at the time of sampling (Kochanowski et al. 2017; Symeonidou et al. 2020; Pettersson et al. 2021b). The type of production could also influence *A. suum* prevalence; in fact, in organic and free-range farms, where animals have continuous access to the outdoor, prevalences could be significantly higher than those found in intensive systems (Eijck and Borgsteede 2005; Delsart et al. 2022).

The recorded prevalence could lead to underestimate the impact of the parasite due to the frequent negativity of the copromicroscopic examination in older pigs following the development of a strong immunity. Indeed, the active immune response can cause a reduction in egg excretion at the end of the fattening cycle but this does not always indicate the absence of the parasite, either at the larval or adult stage, in the host (Symeonidou et al. 2020; Joachim et al. 2021; Delsart et al. 2022). In this regard, a recent study conducted in northern Italy demonstrated a high circulation of *A. suum* by associating serological positivity with the detection of milk spots at slaughter (Vismarra et al. 2023). Thus, despite the finding of low prevalences by copromicroscopy, *A. suum* could be responsible for significant economic losses related to condemnation of livers during slaughter, reduction in weight gain, decreased feed conversion efficiency, and lower meat quality (Boes et al. 2010; Massaglia et al. 2018).

At the farm level, 45.4% (10/22) of farms were positive and, compared with the first sampling session, infection rates and parasite excretion (EPG) were higher at T2; the increase in positivity to *A. suum* could be related to both the long prepatent period of this parasite and the presence of resistant infectious stages from the previous fattening cycle, which might have promoted the infection after housing (Roepstorff et al. 2011; Symeonidou et al. 2020). Further, the use of benzimidazoles after housing in the fattening units, which occurred in most of the sampled farms, does not guarantee protection from infection, as larval stages could escape treatment and the drug administration could be carried out before infection (Joachim et al. 2001). The descriptive analysis showed that 66.7% of farms positive at T1 were also positive at T2, and eggs shed by newly introduced animals into the fattening unit might have contributed to the infections observed at T2. This is supported by another study which highlighted that, at the end of the fattening cycle, prevalence values were higher in pens that were already positive at the beginning of the cycle, probably due to a higher infection pressure (Joachim et al. 2001). The GLMMs demonstrated that two variables were statistically associated with a reduced risk of *A. suum* and nematode infection; in particular, large farms and those applying the all-in/all-out system were at lower risk of infection. In this regard, farm management could greatly influence the presence of nematodes; particularly, herd size would play a central role, as hygienic conditions may be less adequate and biosecurity systems less organised in small herds than in large ones (Kochanowski et al. 2017; Pettersson et al. 2021a). In addition, the application of an all-in/all-out system would allow systematic washing, decontamination and drying between batches, reducing parasite pressure and environmental resistance of eggs before the beginning of a new cycle (Martínez-Pérez et al. 2017; Delsart et al. 2022).

As for *T. suis*, a prevalence of 1.7% was recorded (15/880 positive pigs) and comparable prevalence values, ranging between 0% and 1.4%, were described also in other European countries (Schubnell et al. 2016; Kochanowski et al. 2017; Symeonidou et al. 2020; Pettersson et al. 2021b). It is underlined that in our survey, eggs of *T. suis* were detected only by using the zinc sulphate solution. At the farm level, *T. suis* was found in 6 out of 22 farms (27.3%), and it was shown that a higher prevalence was recorded in farms which did not treat animals with anthelmintics and in those that were positive for *T. suis* as early as the first sampling session. In general, *T. suis* is sporadically found in intensive farms since it is a parasite with a long prepatent period and may not be detected at the time of copromicroscopic analysis (Symeonidou et al. 2020). Further, farm management could greatly influence the farm-level prevalence, as higher rates of *T. suis* positivity were reported in alternative farms, characterised by prolonged access to pastures, although egg excretion level could remain low (Carstensen et al. 2002; Delsart et al. 2022).

Oocysts of *C. suis* were detected in 13.6% (3/22) of the herds and 0.9% of the samples (8/880). It is worth noticing that in one farm, the circulation of *C. suis* was demonstrated at both T1 and T2, probably due to the contamination of the fattening pens by oocysts, which are strongly resistant to ordinary chemicals. Moreover, toltrazuril, that is the target drug for porcine coccidiosis, is only employed on piglets in the farrowing unit and not during the fattening cycle (Straberg and Daugschies 2007; Hinney et al. 2020). In contrast, in the other two positive farms, where seven of the eight positive samples were found, oocysts were detected only in the first sampling session, when the likelihood of infection might be higher (Petterson et al. 2021b). The recorded low prevalence was consistent both with the age of sampled animals, as *C. suis* primarily affects piglets, and with the values reported by other European studies conducted on fatteners, which highlighted prevalence rates ranging from 0% to 3.7% (Koudela and Kucerová 1999; Joachim and Schwarz 2015; Kochanowski et al. 2017; Symeonidou et al. 2020; Pettersson et al. 2021b). The presence of pigs infected by *C. suis* underlines the importance of using appropriate disinfectants to further limit the environmental contamination by coccidian oocysts and reduce the infection pressure throughout the production cycle. Most of the positive samples were reported during the first sampling session; considering the rapid sporulation time and that some effects of *C. suis* infection on young fattening pigs cannot be ruled out, adequate cleaning and disinfection of the fattening site should be ensured (Straberg and Daugschies 2007; Hinney et al. 2020).

Finally, the finding of eggs of *H. diminuta* was interesting since this zoonotic cestode has never been reported in pigs (Panti-May et al 2020). The presence of the cestode eggs in pig faeces could be a case of pseudo-parasitism following the accidental ingestion of carcasses or faeces of infected rodents, i.e. the definitive host of the parasite and suggesting a high circulation of rodents, which could contaminate both breeding units and feed and watering (d’Ovidio et al. 2015). Rodent control at the farm level is crucial because of their central role in the transmission of both pig-specific diseases and parasitic zoonoses and, despite the implementation of proper protocols, the complete exclusion of these animals from pig housing and feed storage facilities could be very difficult (Backhans and Fellström 2012; Andres and Davies 2015). Another plausible option is a case of active parasitism, through the ingestion of intermediate hosts of the parasitic cycle, namely insects of orders Coleoptera, Lepidoptera and Siphonaptera, harbouring cysticercoid larvae. However, further investigation is required to confirm this possibility, associating the egg detection with the presence of adult cestodes in the pig intestine. A recent study highlighted the presence of adults of *H. diminuta* in the intestinal contents of wild boars from Tunisia, although this finding was not associated with the presence of eggs by copromicroscopic analysis (Lahmar et al. 2019). Moreover, some surveys conducted in Asia reported the presence of pig species specific cestodes of the genus *Hymenolepis* and phylogenetically close to *H. diminuta*; however, to date, these parasites have never been described in pigs raised in Europe (Jia et al. 2016; Zhao et al. 2016).

At the farm level, 66.7% of farms positive at T1 were also positive at T2, and this might suggest the possible circulation of the parasite throughout the fattening cycle, either due to the presence of egg-excreting pigs and/or the persistent circulation of rodents and intermediate hosts of the parasitic cycle.

## Conclusions

Data collected in this study provided an overview of the main parasites in fattening pigs in an area of northern Italy characterised by a high density of intensive farms. Gastrointestinal parasites were detected in most of the sampled farms, although with low prevalences, highlighting their persistence and underestimation throughout the fattening cycle. Therefore, appropriate parasite control measures should be developed by acting on possible risk factors related to farm management, e.g. by promoting the continuous flow, characteristic of the all-in/all-out system and developing an integrated approach, which includes appropriate anthelmintics treatment plans and routine faecal monitoring for parasites.

The results obtained in this survey suggest the need for further investigation into the actual impact of these parasites on both animal health and welfare and farm productivity. Further developments should also address the role of *H. diminuta* in pigs, since, to the authors’ knowledge, it has never been detected in this species. Finally, the presence of parasites with zoonotic potential, including both *A. suum* and *H. diminuta*, suggests that all professionals involved in the food chain may be exposed to an increased risk of infection, for which more awareness is needed.

## Supplementary Information

Below is the link to the electronic supplementary material.

Supplementary file1 (DOCX 39 KB)

Supplementary file2 (DOCX 22 KB)

## Data Availability

No datasets were generated or analysed during the current study.
