# Supplementary material for: Epidemiology and distribution of gastrointestinal parasites in fattening pig farms in northern Italy
Source: Parasitol Res. 2024 Aug 22;123(8):307. doi: 10.1007/s00436-024-08320-z (PMC11341578; doi:10.1007/s00436-024-08320-z)
Supplement: Supplementary file 1 — Supplementary file1 (DOCX 39 KB) [file 436_2024_8320_MOESM1_ESM.docx]

**Suppl. Table 1** Detected parasites (eggs or oocysts) and number of positive samples by copromicroscopic analysis for each examined fattening pig farm in northern Italy according to the sampling session (T1= beginning of fattening cycle and T2= end of fattening cycle)

| **Farm code** | **T1 sampling session** | | **T2 sampling session** | |
| --- | --- | --- | --- | --- |
|  | **N° positive samples/total** | **Detected parasites** | **N° positive samples/total** | **Detected parasites** |
| 01 | 0/20 | - | 0/20 | - |
| 02 | 5/20 | *Ascaris suum*,  *Hymenolepis diminuta* | 1/20 | *Hymenolepis diminuta* |
| 03 | 0/20 | - | 0/20 | - |
| 04 | 0/20 | - | 0/20 | - |
| 05 | 0/20 | - | 2/20 | *Ascaris suum* |
| 06 | 2/20 | *Cystoisospora suis* | 7/20 | *Ascaris suum*, *Cystoisospora suis* |
| 07 | 0/20 | - | 1/20 | *Hymenolepis diminuta* |
| 08 | 0/20 | - | 0/20 | - |
| 09 | 2/20 | *Cystoisospora suis* | 0/20 | - |
| 010 | 0/20 | - | 6/20 | *Ascaris suum* |
| 011 | 0/20 | - | 6/20 | *Hymenolepis diminuta*, *Trichuris suis* |
| 012 | 2/20 | *Trichuris suis* | 6/20 | *Ascaris suum*,  *Hymenolepis diminuta*, *Trichuris suis* |
| 013 | 3/20 | *Cystoisospora suis* | 0/20 | - |
| 014 | 1/20 | *Hymenolepis diminuta* | 8/20 | *Ascaris suum* |
| 015 | 0/20 | - | 0/20 | - |
| 016 | 2/20 | *Trichuris suis* | 20/20 | *Ascaris suum*, *Trichuris suis* |
| 017 | 0/20 | - | 0/20 | - |
| 018 | 0/20 | - | 0/20 | - |
| 019 | 1/20 | *Trichuris suis* | 3/20 | *Ascaris suum* |
| 020 | 0/20 | - | 0/20 | - |
| 021 | 4/20 | *Ascaris suum*, *Trichuris suis* | 6/20 | *Ascaris suum* |
| 022 | 4/20 | *Ascaris suum*,  *Hymenolepis diminuta* | 3/20 | *Ascaris suum*,  *Hymenolepis diminuta*, *Trichuris suis* |
