# Supplementary material for: Epidemiology and distribution of gastrointestinal parasites in fattening pig farms in northern Italy
Source: Parasitol Res. 2024 Aug 22;123(8):307. doi: 10.1007/s00436-024-08320-z (PMC11341578; doi:10.1007/s00436-024-08320-z)
Supplement: Supplementary file 2 — Supplementary file2 (DOCX 22 KB) [file 436_2024_8320_MOESM2_ESM.docx]

**Suppl. Table 2** Management characteristics of fattening pig farms recruited in northern Italy and prevalence values of detected parasites at T2 sampling session for each considered variable

| **Detected parasites** | **Variable** | **Category** | **N° of positive samples/total at T2 sampling session** | **Prevalence % (95% CI^a^) at T2 sampling session** | **N° of positive farms/total at T2 sampling session** | **Prevalence % (95% CI) at T2 sampling session** |
| --- | --- | --- | --- | --- | --- | --- |
| *A. suum* | All-in/All-out | Yes | 28/320 | 8.7 (5.9-12.4) | 6/16 | 37.5 (15.2-64.6) |
|  |  | No | 29/120 | 24.1 (16.8-32.8) | 3/6 | 50 (11.8-88.2) |
|  | Antiparasitic treatment | Yes | 36/360 | 10 (7.1-13.6) | 7/18 | 38.9 (17.3-64.2) |
|  |  | No | 21/80 | 26.2 (17-37.3) | 2/4 | 50 (6.8-93.2) |
|  | Farm size | <1900 animals | 49/280 | 17.5 (13.2-22.5) | 8/14 | 57.1 (28.9-82.3) |
|  |  | ≥ 1900 animals | 8/160 | 5 (2.2-9.6) | 1/8 | 12.5 (0.3-52.6) |
|  | Outdoor access | Yes | 22/240 | 9.2 (5.8-13) | 4/12 | 33.3 (9.9-65.1) |
|  |  | No | 35/200 | 17.5 (12.5-23.5) | 5/10 | 50 (18.7-81.3) |
|  | Type of floor | Full | 34/240 | 14.2 (10-19.2) | 4/12 | 33.3 (9.9-65.1) |
|  |  | Mixed | 4/40 | 10 (2.8-23.7) | 1/2 | 50 (1.3-98.7) |
|  |  | Slatted | 19/160 | 11.9 (7.3-17.9) | 4/8 | 50 (15.7-84.3) |
|  | Farm positivity at T1 sampling session for *A. suum* | Yes | 8/60 | 13.3 (5.9-24.6) | 2/3 | 66.7 (9.4-99.2) |
|  |  | No | 49/380 | 12.9 (9.7-16.7) | 7/19 | 36.8 (16.3-61.6) |
| *C. suis* | All-in/All-out | Yes | 1/320 | 0.3 (0-1.7) | 1/16 | 6.2 (0.2-30.2) |
|  |  | No | 0/120 | 0 (-) | 0/6 | 0 (-) |
|  | Antiparasitic treatment | Yes | 1/360 | 0.2 (0-1.5) | 1/18 | 5.6 (0.1-27.3) |
|  |  | No | 0/80 | 0 (-) | 0/4 | 0 (-) |
|  | Farm size | <1900 animals | 1/280 | 0.4 (0-2) | 1/14 | 7.1 (0.2-33.9) |
|  |  | ≥ 1900 animals | 0/160 | 0 (-) | 0/8 | 0 (-) |
|  | Outdoor access | Yes | 1/240 | 0.4 (0-2.3) | 1/12 | 8.3 (0.2-38.9) |
|  |  | No | 0/200 | 0 (-) | 0/10 | 0 (-) |
|  | Type of floor | Full | 1/240 | 0.4 (0-2.3) | 1/12 | 8.3 (0.2-38.9) |
|  |  | Mixed | 0/40 | 0 (-) | 0/2 | 0 (-) |
|  |  | Slatted | 0/160 | 0 (-) | 0/8 | 0 (-) |
|  | Farm positivity at T1 sampling session for *C. suis* | Yes | 1/60 | 1.7 (0-8.9) | 1/3 | 33.3 (0.8-90.6) |
|  |  | No | 0/380 | 0 (-) | 0/19 | 0 (-) |
| *H. diminuta* | All-in/All-out | Yes | 7/320 | 2.2 (0.9-4.4) | 4/16 | 25 (7.3-52.4) |
|  |  | No | 1/120 | 0.8 (0-4.6) | 1/6 | 16.7 (0.4-64.1) |
|  | Antiparasitic treatment | Yes | 7/360 | 1.9 (0.7-4) | 4/18 | 22.2 (6.4-47.6) |
|  |  | No | 1/80 | 1.2 (0-6.8) | 1/4 | 25 (0.6-80.6) |
|  | Farm size | <1900 animals | 7/280 | 2.5 (1-5.1) | 4/14 | 28.6 (8.4-58.1) |
|  |  | ≥ 1900 animals | 1/160 | 0.6 (0-3.4) | 1/8 | 12.5 (0.3-52.6) |
|  | Outdoor access | Yes | 6/240 | 2.5 (0.9-5.4) | 3/12 | 25 (5.5-57.2) |
|  |  | No | 2/200 | 1 (0.1-3.6) | 2/10 | 20 (2.5-55.6) |
|  | Type of floor | Full | 6/240 | 2.5 (0.9-5.4) | 3/12 | 25 (5.5-57.2) |
|  |  | Mixed | 1/40 | 2.5 (0-13.2) | 1/2 | 50 (1.3-98.7) |
|  |  | Slatted | 1/160 | 0.6 (0-3.4) | 1/8 | 12.5 (0.3-52.6) |
|  | Farm positivity at T1 sampling session for *H. diminuta* | Yes | 2/60 | 3.3 (0.4-11.5) | 2/3 | 66.7 (9.4-99.2) |
|  |  | No | 6/380 | 1.2 (0.6-3.4) | 3/19 | 15.8 (3.4-39.6) |
| *T. suis* | All-in/All-out | Yes | 4/320 | 1.2 (0.3-3.2) | 2/16 | 12.5 (1.5-38.3) |
|  |  | No | 5/120 | 4.2 (1.4-9.5) | 2/6 | 33.3 (4.3-77.7) |
|  | Antiparasitic treatment | Yes | 4/360 | 1.1 (0.3-2.8) | 2/18 | 11.1 (1.4-34.7) |
|  |  | No | 5/80 | 6.2 (2.1-14) | 2/4 | 50 (6.8-93.2) |
|  | Farm size | <1900 animals | 9/280 | 3.2 (1.5-6) | 4/14 | 28.6 (8.4-58.1) |
|  |  | ≥ 1900 animals | 0/160 | 0 (-) | 0/8 | 0 (-) |
|  | Outdoor access | Yes | 4/240 | 1.7 (0.5-4.2) | 2/12 | 16.7 (2.1-48.4) |
|  |  | No | 5/200 | 2.5 (0.8-5.7) | 2/10 | 20 (2.5-55.6) |
|  | Type of floor | Full | 7/240 | 2.9 (1.2-5.9) | 3/12 | 25 (5.5-57.2) |
|  |  | Mixed | 2/40 | 5 (0.6-16.9) | 1/2 | 50 (1.3-98.7) |
|  |  | Slatted | 0/160 | 0 (-) | 0/8 | 0 (-) |
|  | Farm positivity at T1 sampling session for *T. suis* | Yes | 6/80 | 7.5 (2.8-15.6) | 2/4 | 50 (6.8-93.2) |
|  |  | No | 3/360 | 0.8 (0.1-2.4) | 2/18 | 11.1 (1.4-34.7) |

^a^Confidence Interval
